# Insanity and Its Treatment

**Published:** 1886-08

**Authors:** 


INSANITY AND ITS TREATMENT. Lectures on the Treatment, Medical and Legal, of Insane Patients. By G. Fielding Blandford, M. D., Oxon. Fellow of the Royal College of Physicians in London; late Lecturer on Physiological Medicine at the School of St. George's Hospital, London ; third edition; together with Types of Insanity. An Illustrated Guide in the Physical Diagnosis of Mental Disease, by Allan McLane Hamilton, M. D., one of the Consulting Physicians to the Insane Asylums of New York City, and the Hudson River State Hospital for the Insane, etc. New York: William Wood & Co., 56 and 58 LaFayette Place. 1886.

This work is made up of lectures delivered by the author at the schools of St. George's Hospital, London. The author, in his introductory, says if there be one branch of the great study of medicine which more than another deserves to be called an art and a mystery, it is the treatment and investigation of insanity. The treatment is an art which, during the present century, has advanced in a degree not inferior to other arts, and in which by practice and example we may hope to attain skill, as in surgery or midwifery, but the disorder which we call insanity is a mystery not yet unraveled.

The author has done much to unravel this mystery, as every one will conclude who reads this valuable book.

